# Parvalbumin Interneuron Dysfunction in a Thalamo-Prefrontal Cortical Circuit in *Disc1* Locus Impairment Mice

**DOI:** 10.1523/ENEURO.0496-19.2020

**Published:** 2020-03-02

**Authors:** Kristen Delevich, Hanna Jaaro-Peled, Mario Penzo, Akira Sawa, Bo Li

**Affiliations:** 1 Cold Spring Harbor Laboratory, Cold Spring Harbor, NY 11724; 2 Watson School of Biological Sciences, Cold Spring Harbor Laboratory, Cold Spring Harbor, NY 11724; 3 National Institute of Mental Health, Bethesda, MD 20892; 4 Department of Psychiatry, Johns Hopkins University School of Medicine, Baltimore, MD 21287

**Keywords:** disrupted-in-schizophrenia-1, feedforward inhibition, mediodorsal thalamus, parvalbumin interneurons, prefrontal cortex

## Abstract

Altered cortical excitation-inhibition (E-I) balance resulting from abnormal parvalbumin interneuron (PV IN) function is a proposed pathophysiological mechanism of schizophrenia and other major psychiatric disorders. Preclinical studies have indicated that *disrupted-in-schizophrenia-1* (*Disc1*) is a useful molecular lead to address the biology of prefrontal cortex (PFC)-dependent cognition and PV IN function. To date, PFC inhibitory circuit function has not been investigated in depth in *Disc1* locus impairment (LI) mouse models. Therefore, we used a *Disc1* LI mouse model to investigate E-I balance in medial PFC (mPFC) circuits. We found that inhibition onto layer 2/3 excitatory pyramidal neurons in the mPFC was significantly reduced in *Disc1* LI mice. This reduced inhibition was accompanied by decreased GABA release from local PV, but not somatostatin (SOM) INs, and by impaired feedforward inhibition (FFI) in the mediodorsal thalamus (MD) to mPFC circuit. Our mechanistic findings of abnormal PV IN function in a *Disc1* LI model provide insight into biology that may be relevant to neuropsychiatric disorders including schizophrenia.

## Significance Statement

A popular theory suggests that dysregulation of fast-spiking parvalbumin interneurons (PV INs) and elevated excitation-inhibition (E-I) balance contribute to the pathophysiology of various psychiatric disorders. Previous studies suggest that genetic perturbations of the *disrupted-in-schizophrenia-1* (*Disc1*) gene affect prefrontal cortex (PFC)-dependent cognition and PV IN function, but synaptic and circuit physiology data are lacking. Here, we provide evidence that the presynaptic function of PV INs in the medial PFC (mPFC) is altered in *Disc1* LI mice and that E-I balance is elevated within a thalamofrontal circuit known to be important for cognition. These findings may contribute to our understanding of the biology that gives rise to cognitive symptoms in a range of neuropsychiatric disorders.

## Introduction

Parvalbumin interneurons (PV INs) provide powerful somatic inhibition to excitatory pyramidal neurons and regulate excitation-inhibition (E-I) balance (Isaacson and Scanziani, 2011). Prefrontal PV INs are implicated in working memory (WM) function (Cardin et al., 2009; Fries, 2009; Sohal et al., 2009; Murray et al., 2015; Ferguson and Gao, 2018b) and have emerged from human postmortem studies as a key node of interest in the pathophysiology of schizophrenia (Beasley and Reynolds, 1997; Hashimoto et al., 2003; Lewis et al., 2012). Therefore, dysregulation of E-I balance via altered PV IN function is a potential pathophysiological mechanism of particular relevance to cognitive symptoms of neuropsychiatric diseases, including schizophrenia. Cognitive impairment is seen in first-degree relatives of individuals with a range of major mental illnesses (Cannon et al., 2000; Myles-Worsley and Park, 2002; Snitz et al., 2006), suggesting that these processes are partly heritable and may be better understood through investigation of promising genetic and molecular leads.

A translocation in the gene *disrupted-in-schizophrenia-1* (*Disc1*) was first reported in a Scottish pedigree as a rare but penetrant genetic risk factor that may account for a wide range of major mental illnesses such as depression and schizophrenia (Millar et al., 2000). This suggests that biological pathway(s) involving the multifunctional hub protein DISC1 contribute to cognitive and behavioral dimensions that are disrupted in neuropsychiatric illnesses (Niwa et al., 2016). While DISC1 is not a common genetic variant associated with schizophrenia in large population samples (Schizophrenia Working Group of the Psychiatric Genomics C, 2014), it can serve as a molecular lead to study the biology underlying important constructs/dimensions that are relevant to major mental illness (Niwa et al., 2016). Work in mouse models has revealed the importance of DISC1 in neurodevelopment (Kamiya et al., 2005; Ishizuka et al., 2007, 2011; Mao et al., 2009; Niwa et al., 2010), synaptic function (Hayashi-Takagi et al., 2010; Wang et al., 2011; Maher and LoTurco, 2012; Sauer et al., 2015; Seshadri et al., 2015; Wei et al., 2015), and cognitive processing (Brandon and Sawa, 2011). WM impairments are consistently reported across *Disc1* mouse models (Koike et al., 2006; Clapcote et al., 2007; Li et al., 2007; Kvajo et al., 2008; Lipina et al., 2010; Niwa et al., 2010; Brandon and Sawa, 2011; Lee et al., 2013). Furthermore, a variety of *Disc1* mouse models exhibit reduced prefrontal PV expression (Hikida et al., 2007; Shen et al., 2008; Ibi et al., 2010; Niwa et al., 2010; Ayhan et al., 2011; Lee et al., 2013). While these data are suggestive that PV INs may be particularly affected by *Disc1* perturbation, evidence from synaptic and circuitry physiology is lacking.

Here, we investigated the synaptic and circuit level function of PV INs within medial prefrontal cortex (mPFC) circuits of mice heterozygous for the *Disc1* locus impairment (LI) allele, in which the majority of *Disc1* isoforms are abolished (Seshadri et al., 2015; Shahani et al., 2015). We found that *Disc1* LI was associated with elevated E-I balance and altered PV IN function in mPFC circuits relevant to cognition.

## Materials and Methods

### Animals

Mice were group housed under a 12/12 h light/dark cycle (9 A.M. to 9 P.M. light), with food and water freely available. Both male and female mice were used. All procedures involving animals were approved by the Institutional Animal Care and Use Committee of Cold Spring Harbor Laboratory and conducted in accordance with the National Institutes of Health guidelines. The *PV-Cre* (http://jaxmice.jax.org/strain/008069.html), *SOM-Cre* (http://jaxmice.jax.org/strain/013044.html), and *Ai14* (https://www.jax.org/strain/007914) mice were described previously (Hippenmeyer et al., 2005; Madisen et al., 2010; Taniguchi et al., 2011). We previously generated the *Disc1* LI mice, which harbor a deletion (6.9 kb) encompassing the first three exons of the *Disc1* gene (Seshadri et al., 2015). The majority of DISC1 isoforms are abolished in mice homozygous for the *Disc1* LI allele (Seshadri et al., 2015), and in the current study we used mice that harbored one *Disc1* LI allele (+/−). All mice were bred onto C57BL/6 background for at least five generations.

### Viral vectors

Adeno-associated virus (AAV) vectors AAV-CAG-ChR2(H134R)-YFP and AAV-eF1a-DIO-ChR2(H134R)-YFP were produced as AAV2/9 serotype by the University of North Carolina Vector Core and have been previously described (Zhang et al., 2007; Delevich et al., 2015). All viral vectors were stored in aliquots at −80°C until use.

### Stereotaxic surgery

Mice aged postnatal day (P)40–56 were used for all surgeries. Unilateral viral injections were performed using previously described procedures (Li et al., 2013) at the following stereotaxic coordinates: MD: –1.58 mm from bregma, 0.44 mm lateral from midline, and 3.20 mm ventral from cortical surface; dorsal mPFC: 1.94 mm from bregma, 0.34 mm lateral from midline, and 0.70 mm ventral from cortical surface. Surgical procedures were standardized to minimize the variability of AAV injections. To ensure minimal leak into surrounding brain areas, injection pipettes remained in the brain for ∼5 min after injection before being slowly withdrawn. The final volume for AAV-CAG-ChR2(H134R)-YFP injected into MD was 0.3–0.35 μl, and for AAV-eF1a-DIO-ChR2(H134R)-YFP injected into dorsal mPFC was 0.5 μl. The titer for the viruses was ∼10^12^ viral particles/ml. For experiments in which mPFC inhibitory INs were optogenetically stimulated (Fig. 2) mice were injected at P56 and approximately two weeks were allowed for viral expression before recording. For experiments in which MD axons within mPFC were optogenetically stimulated (Figs. 3, 5) mice were injected at P40–45 and approximately four weeks were allowed for viral expression before recording. For each of these experiments, littermates were injected and recorded at the same age to control for expression duration between genotypes.

### Electrophysiology

Mice were anaesthetized with isoflurane and decapitated, whereupon brains were quickly removed and immersed in ice-cold dissection buffer (110.0 mM choline chloride, 25.0 mM NaHCO_3_, 1.25 mM NaH_2_ PO_4_, 2.5 mM KCl, 0.5 mM CaCl_2_, 7.0 mM MgCl_2_, 25.0 mM glucose, 11.6 mM ascorbic acid, and 3.1 mM pyruvic acid, gassed with 95% O_2_ and 5% CO_2_). Coronal slices (300 μm in thickness) containing mPFC were cut in dissection buffer using a HM650 Vibrating Microtome (Thermo Fisher Scientific), and were subsequently transferred to a chamber containing artificial CSF (ACSF; 118 mM NaCl, 2.5 mM KCl, 26.2 mM NaHCO_3_, 1 mM NaH_2_ PO_4_, 20 mM glucose, 2 mM MgCl_2_, and 2 mM CaCl_2_, at 34°C, pH 7.4, gassed with 95% O_2_ and 5% CO_2_). After ∼30 min of recovery time, slices were transferred to room temperature and were constantly perfused with ACSF.

The internal solution for voltage-clamp experiments contained 140 mM potassium gluconate, 10 mM HEPES, 2 mM MgCl_2_, 0.05 mM CaCl_2_, 4 mM MgATP, 0.4 mM Na_3_GTP, 10 mM Na_2_-phosphocreatine, 10 mM BAPTA, and 6 mM QX-314 (pH 7.25, 290 mOsm). Electrophysiological data were acquired using pCLAMP 10 software (Molecular Devices). Miniature IPSCs (mIPSCs) were recorded in the presence of tetrodotoxin (1 μM), APV (100 μM), and CNQX (5 μM). Miniature EPSCs (mEPSCs) were recorded in the presence of tetrodotoxin (1 μM) and picrotoxin (100 μM). Data were analyzed using Mini Analysis Program (Synaptosoft). For the mIPSCs and mEPSCs, we analyzed the first 300 and 250 events, respectively, for each neuron. The parameters for detecting mini events were kept consistent across neurons, and data were quantified blind to genotype.

To evoke synaptic transmission by activating channelrhodopsin-2 (ChR2), we used a single-wavelength LED system (λ = 470 nm; CoolLED) connected to the epifluorescence port of the Olympus BX51 microscope. To restrict the size of the light beam for focal stimulation, a built-in shutter along the light path in the BX51 microscope was used. Light pulses of 0.5–1 ms triggered by a transistor-transistor logic (TTL) signal from the Clampex software (Molecular Devices) were used to evoke synaptic transmission. The light intensity at the sample was ∼0.8 mW/mm^2^. Electrophysiological data were acquired and analyzed using pCLAMP 10 software (Molecular Devices). IPSCs were recorded at 0 mV holding potential in the presence of 5 μM CNQX and 100 μM AP-5. Light pulses were delivered once every 10 s, and a minimum of 30 trials were collected. In paired-pulse recordings, 2 light pulses separated by 50, 100, or 150 ms were delivered. In cases that the first IPSC did not fully decay to baseline before the onset of the second IPSC, the baseline of the second IPSC was corrected before the peak was measured. To measure the kinetics of the IPSCs, averaged sweeps collected at the 150-ms interval were normalized, and the decay time constant and half-width were measured using automated procedures in the AxoGraph X 1.5.4 software.

To determine IPSC reversal potential (E_IPSC_), IPSCs were recorded at varying holding potentials (20-mV steps) in the presence of CNQX (5 μM) and AP-5 (100 μM) to block AMPA receptors and NMDA receptors, respectively. IPSC amplitude was measured, and a linear regression was used to calculate the best-fit line, and the x-intercept was used as the E_IPSC_. Under our recording conditions, the E_IPSC_ was ∼–60 mV. Therefore, in the excitation/inhibition ratio (E/I) experiments, we recorded EPSCs at –60 mV and IPSCs at 0 mV holding potential. The only drug used for the E/I experiments was AP-5 (100 μM). In these experiments we used the same light intensity for evoking both IPSCs and EPSCs. In addition, we used similar stimulation regime for WT and *Disc1* LI mice, such that the peak amplitudes of IPSCs were comparable between genotypes.

For the experiments in which we optogenetically stimulated the MD axons in the mPFC, mice were excluded if the extent of infection in the MD was too large and leaked into surrounding brain regions. Rodent MD lacks INs; therefore all ChR2-infected neurons are expected to be relay projection neurons (Kuroda et al., 1998).

**Figure 1.**
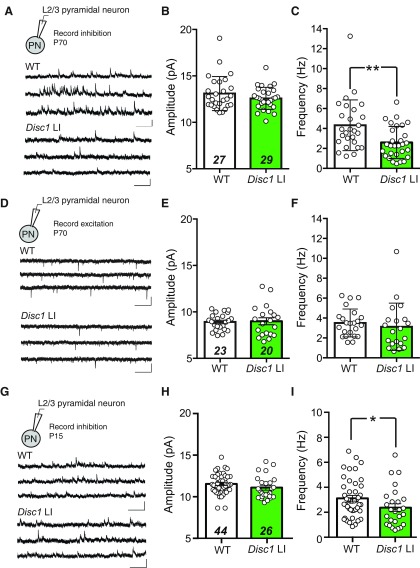
Reduced inhibitory synaptic transmission onto L2/3 pyramidal neurons in the mPFC of adult and juvenile *Disc1* LI mice. ***A***, Recording configuration and sample mIPSC traces recorded from L2/3 PNs in the mPFC of WT (upper) and *Disc1* LI (lower) mice at ∼P70. ***B***, mIPSC amplitude (WT, *n* = 27 cells; *Disc1* LI, *n* =* *29 cells). ***C***, mIPSC frequency (WT, *n* = 27 cells; *Disc1* LI, *n* = 29 cells; **p <* *0.01, Mann–Whitney *U* test). ***D***, Recording configuration and sample mEPSC traces recorded from L2/3 PNs in the mPFC of WT (upper) and *Disc1* LI (lower) mice at ∼P70. ***E***, mEPSC amplitude (WT, *n* = 23 cells; *Disc1* LI, *n* = 20 cells). ***F***, mEPSC frequency (WT, *n* = 23 cells; *Disc1* LI, *n* = 20 cells). ***G***, Recording configuration and sample mIPSC traces recorded from L2/3 PNs in the mPFC of WT (upper) and *Disc1* LI (lower) mice at ∼P15. mIPSC (***H***) amplitude (WT, *n* = 44 cells; *Disc1* LI, *n* = 26 cells; *p *=* *0.12, *t* test) and (***I***) frequency (WT, *n* = 44 cells; *Disc1* LI, *n* = 26 cells; *p <* *0.05, Mann–Whitney *U* test). All scale bars represent 20 pA, 500 ms. Bar graphs indicate median ± interquartile range (***B***, ***C***, ***F***, ***I***) or mean ± SEM (***E***, ***H***), as appropriate.

**Figure 2.**
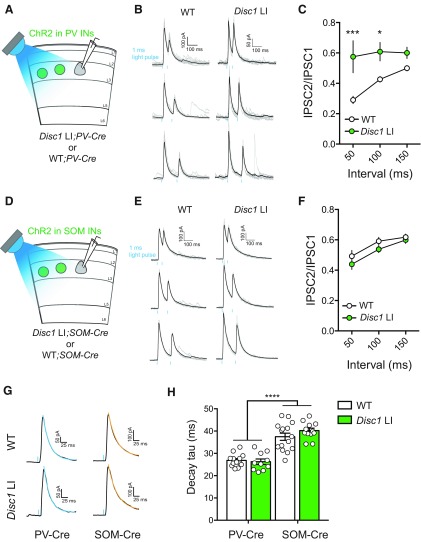
Altered presynaptic function of PV but not SOM INs in the mPFC of *Disc1* LI mice. ***A***, Schematic of the experimental configuration. ***B***, Sample traces of PV IN-mediated IPSCs recorded from WT (left panel) or *Disc1* LI (right panel) mice. Paired light pulses (1-ms duration; blue bars) were delivered at an interval of 50 ms (top), 100 ms (middle), or 150 ms (bottom). ***C***, Quantification of PPR for each genotype (WT, *n* = 13 cells; *Disc1* LI, *n* = 10 cells); *p <* *0.05, ***p <* *0.001, two-way RM ANOVA followed by Sidak’s test. ***D***, Schematic of the experimental configuration. ***E***, Sample traces of SOM IN-mediated IPSCs recorded from WT (left panel) or *Disc1* LI (right panel) mice. Paired light pulses (1-ms duration; blue bars) were delivered at an interval of 50 ms (top), 100 ms (middle), or 150 ms (bottom). ***F***, Quantification of PPR for each genotype (WT, *n* = 15 cells, *Disc1* LI, *n* = 12 cells). ***G***, Sample IPSC traces evoked by optogenetic stimulation of PV or SOM INs. Colored lines indicate exponential fits to the decays of the IPSCs. ***H***, Quantification of IPSC decay tau; ****p <* *0.0001, *t* test. Data in ***C***, ***F***, ***H*** are presented as mean ± SEM.

**Figure 3.**
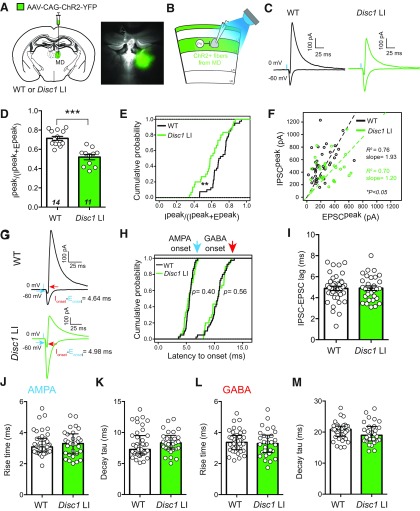
Reduced FFI in the MD–mPFC circuit in *Disc1* LI mice. ***A***, ***B***, Schematics of the experimental configuration. The right panel of ***A*** is an image of a brain section from a mouse used in electrophysiological recording, showing MD infected with AAV-CAG-ChR2-YFP. ***C***, Representative traces of evoked EPSC (recorded at –60 mV) and IPSC (recorded at 0 mV) from L3 PNs. ***D***, To estimate the relative recruitment of disynaptic FFI versus monosynaptic excitation, we divided peak IPSC (I^peak^) by the sum of peak IPSC and peak EPSC (I^peak^ + E^peak^). WT, *N* = 14 mice, *Disc1* LI, *N* = 11 mice; ***p <* *0.001, *t* test. ***E***, Same as in ***D***, except that the cumulative probability distributions of the values for individual neurons are shown. WT, *n* = 40 cells, *Disc1* LI, *n* = 30 cells; **p <* *0.01, Kolmogorov–Smirnov test. ***F***, Scatter plot showing the peak amplitudes of IPSC and EPSC for individual neurons. Each circle represents one neuron (WT, *n* = 30 cells; *Disc1* LI, *n* = 40 cells). Dashed lines are linear regression lines for neurons in WT mice and *Disc1* LI mice. The slopes of the regression lines significantly differed at the 0.95 confidence level (*p <* *0.05). ***G***, Sample traces of IPSC (recorded at 0 mV) and EPSC (recorded at –60 mV) recorded from L2/3 PNs in response to light-stimulation (blue bars) of MD axons. The latency to onset was measured from the time the light stimulus was triggered to the 10% EPSC (blue arrow) or IPSC (red arrow) rise time. Note that IPSC rise time was calculated from the peak of the inward current recorded at 0 mV. ***H***, Cumulative probability distributions for EPSC latency to onset (left) and IPSC latency to onset (right; EPSC, WT, *n* = 40 cells, *Disc1* LI, *n* = 30 cells, *p *=* *0.40; IPSC, WT, *n* = 40 cells, *Disc1* LI, *n* = 30 cells, *p *=* *0.56; Kolmogorov–Smirnov test). ***I***, Quantification of IPSC–EPSC lag, calculated as the difference in the latency to onset between the IPSC and the EPSC of each neuron (see also ***G***; WT, *n* = 40 cells, *Disc1* LI, *n* = 30 cells; *p *>* *0.05, *t* test). ***J***, Quantification of the 10–90% EPSC rise time and decay tau (***K***; WT, *n* = 40 cells, *Disc1* LI, *n* = 30 cells; *p *>* *0.05, Mann–Whitney *U* test). ***L***, Quantification of the 10–90% IPSC rise time and decay tau (*M*; WT, *n* = 40 cells, *Disc1* LI, *n* = 28 cells; *p *>* *0.05, *t* test). Data are presented as median ± interquartile range (***J***, ***K***) or mean ± SEM (***D***, ***L***, ***M***).

**Figure 4.**
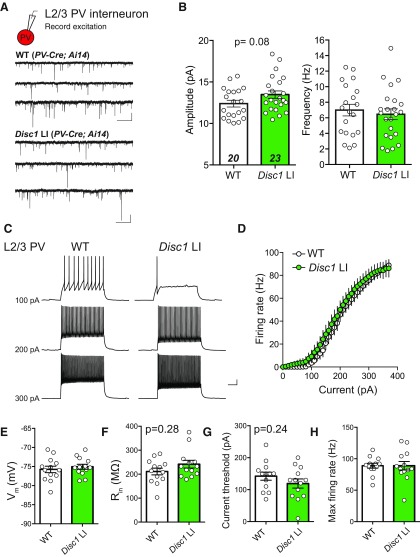
mEPSCs and intrinsic properties of PV INs are not altered in *Disc1* LI mice. ***A***, Recording configuration and sample mEPSC traces recorded from PV INs in WT (upper) and *Disc1* LI mice (lower). ***B***, Mean mEPSC amplitude (left) and median frequency (right; *n* = 20, 23 cells/genotype; *N* = 4 mice/genotype). ***C***, Sample traces from whole-cell current clamp recording of L2/3 PV IN in WT (left) and *Disc1* LI mouse in response to current injections. ***D***, Input-output curve showing average firing rate of PV INs in response to current injection in WT versus *Disc1* LI. ***E***, Resting membrane potential. ***F***, Input resistance. ***G***, Current threshold required to elicit spiking. ***H***, Maximum firing rate. Data in ***D***, ***E***, ***H*** shown as mean ± SEM. Data in ***F***, ***G*** shown as median ± interquartile range.

The latency and 10–90% rise time of EPSCs and IPSCs were calculated from either the averaged trace or individual sweeps for each cell using automated procedures in the AxoGraph X 1.5.4 software. ESPC and IPSC onset latency was calculated as the time from stimulation onset to 10% rise time, with EPSC-IPSC lag calculated as the difference. The 10% rise time has been reported to be a more reliable measure of delay to onset, as it minimizes the contribution of EPSC and IPSC rise time differences that are reflected in the time to peak (Mittmann et al., 2005). Some of the control data from WT mice used for comparing with *Disc1* LI mice (appearing in Figs. 3, 4) were previously reported in Delevich et al., 2015, (Figs. 1, 2, and 4).

### Data analysis and statistics

All statistical tests were performed using Origin 9.0 (Origin-Lab) or GraphPad Prism 6.0 (GraphPad Software) software. All data were tested for normality using the D’Agostino–Pearson omnibus normality test to guide the selection of parametric or non-parametric statistical tests. Data are presented as mean ± SEM or median ± interquartile range as indicated. For parametric data, a two-tailed *t* test or two-way ANOVA was used, with a *post hoc* Sidak’s test for multiple comparisons. For non-parametric data, a two-tailed Mann–Whitney *U* test was used; *p *<* *0.05 was considered significant. A summary of the statistical analyses performed can be found in Table 1.

**Table 1:** Statistical tests and significance threshold used for each comparison

**Data structure**	**Statistical test, *post hoc***	**Significance threshold**
^a^Non-normal distribution	Two-tailed Mann–Whitney *U* test	*p* < 0.05
^b^Non-normal distribution	Two-tailed Mann–Whitney *U* test	*p* < 0.05
^c^Non-normal distribution	Two-tailed Mann–Whitney *U* test	*p* < 0.05
^d^Normal distribution	Two-tailed unpaired *t* test	*p* < 0.05
^e^Normal distribution	Two-tailed unpaired *t* test	*p* < 0.05
^f^Non-normal distribution	Two-tailed Mann–Whitney *U* test	*p* < 0.05
^g^Normal distribution	Two-way RM ANOVA with *post hoc* Sidak’s test	*p* < 0.05
^h^Normal distribution	Two-way RM ANOVA with *post hoc* Sidak’s test	*p* < 0.05
^i^Normal distribution	Two-way ANOVA	*p* < 0.05
^j^Normal distribution	Two-tailed unpaired *t* test	*p* < 0.05
^k^Normal distribution	Two-tailed unpaired *t* test	*p* < 0.05
^l^Normal distribution	Two-tailed unpaired *t* test	*p* < 0.05
^m^Normal distribution	Two-tailed unpaired *t* test	*p* < 0.05
^n^Normal distribution	Two-tailed unpaired *t* test	*p* < 0.05
^o^Normal distribution	Two-tailed unpaired *t* test	*p* < 0.05
^p^Non-normal distribution	Wilcoxon matched-pairs signed ranks test	*p* < 0.05
^q^Non-normal distribution	Wilcoxon matched-pairs signed ranks test	*p* < 0.05
^r^Non-normal distribution	Two-tailed Mann–Whitney *U* test	*p* < 0.05
^s^Non-normal distribution	Two-tailed Mann–Whitney *U* test	*p* < 0.05

## Results

### Inhibitory synaptic transmission is reduced in adult *Disc1* LI mice

As a first estimation of inhibitory drive in the mPFC, we recorded mIPSCs onto L2/3 PNs in the dorsal anterior cingulate cortex (dACC) subregion of the mPFC in adult mice (P70). We found that compared with wild-type (WT) littermates, *Disc1* LI mice had significantly reduced mIPSC frequency (WT, 3.75 ± 3.25 Hz, *n* = 27 cells, *N* = 6; *Disc1* LI, 2.27 ± 2.72 Hz, *n* = 29 cells, *N* = 5; *U *=* *217.0, ^a^*p *<* *0.01, Mann–Whitney *U* test), but not amplitude (WT, 12.5 ± 2.48 pA, *n* = 29 cells, *N* = 6; *Disc1* LI, 12.52 ± 1.51 pA, *n* = 27 cells, *N* = 5; *U *=* *351.0, ^b^*p *=* *0.51, Mann–Whitney *U* test; Fig. 1*A–C*). The two groups did not differ in measures of mEPSCs (frequency: WT, 3.47 ± 1.97 Hz, *n* = 23 cells, *N* = 4; *Disc1* LI, 2.79 ± 2.4 Hz, *n* = 20 cells, *N* = 5; *U *=* *173.0, ^c^*p *=* *0.17, Mann–Whitney *U* test; amplitude: WT, 8.91 ± 0.18 pA; *Disc1* LI, 8.99 ± 0.37 pA, *t*_(41)_ = 0.18, ^d^*p *=* *0.86, *t* test; Fig. 1*D–F*). Notably, we found that the frequency (but not amplitude) of mIPSCs recorded from dACC L2/3 PNs in *Disc1* LI mice was lower compared to their WT littermates at preweanling (∼P15) age (amplitude: WT, *n* = 44 cells; *Disc1* LI, *n* = 26 cells; ^e^*p *=* *0.12, *t* test; frequency: WT, *n* = 44 cells; *Disc1* LI, *n* = 26 cells; ^f^*p *<* *0.05, Mann–Whitney *U* test; Fig. 1*G–I*). These data indicate that the inhibitory synaptic transmission is selectively impaired in the mPFC of *Disc1* LI mice, and that this impairment manifests early in postnatal development.

### Altered presynaptic function of PV INs in *Disc1* LI mice

A reduction in mIPSC frequency could result from a decrease in synaptic transmission from one or more inhibitory IN populations. To investigate the source of reduced inhibitory drive onto L2/3 PNs in the mPFC of *Disc1* LI mice, we sought to examine the IPSCs originating from either PV or somatostatin (SOM) INs. To this end, we selectively expressed ChR2, the light-gated cation channel (Zhang et al., 2006), in PV or SOM INs by injecting the mPFC of *Disc1* LI*; PV-Cre* or *Disc1* LI*; SOM-Cre* mice, as well as their WT littermates, with an AAV expressing ChR2 in a Cre-dependent manner (AAV-DIO-ChR2(H134R)-YFP). After viral expression had reached sufficient levels, we prepared acute brain slices and recorded light-evoked IPSCs onto L2/3 PNs in mPFC (Fig. 2*A*,*D*). We delivered paired light pulses (pulse duration 1 ms) with an interpulse interval of 50, 100, or 150 ms, and measured the ratio of the peak amplitude of the second IPSC over that of the first (IPSC_2_/IPSC_1_), also known as paired-pulse ratio (PPR; Fig. 2*B*,*E*). A similar technique was previously used to interrogate presynaptic GABA release from PV INs (Chu et al., 2012).

We found that the PPR of GABAergic transmission between PV INs and L2/3 PNs was significantly increased in the *Disc1* LI mice compared to their WT littermates at the 50- and 100-ms interpulse intervals (WT, *n* = 13 cells; *Disc1* LI*, n* = 10 cells; interval: *F*_(2,42)_ = 6.77, ^g^*p *<* *0.01; genotype: *F*_(1,21)_ = 10.77, *p *<* *0.01; interaction: *F*_(2,42)_ = 3.92, *p *<* *0.05; two-way repeated-measures (RM) ANOVA followed by Sidak’s tests; Fig. 2*C*), suggesting that GABA release from PV INs is impaired. In contrast, the PPR of GABAergic synaptic transmission from SOM INs to L2/3 PNs did not differ between genotypes (WT, *n* = 15 cells, *Disc1* LI, *n* = 12 cells; interval: *F*_(2,50)_ = 24.88, ^h^*p *<* *0.0001; genotype: *F*_(1,25)_ = 1.64, *p *=* *0.21; interaction *F*_(2,50)_ = 0.47, *p *=* *0.63, two-way RM ANOVA; Fig. 2*F*). SOM-evoked IPSCs displayed significantly slower decay kinetics than PV-evoked IPSCs (Fig. 2*G*,*H*), consistent with previous reports (Koyanagi et al., 2010; Ma et al., 2012). No differences in IPSC kinetics were observed between *Disc1* LI mice and their WT littermates (cell type: *F*_(1,46)_ = 90.82, ^i^*p *<* *0.0001; genotype: *F*_(1,46)_ = 0.678, *p *=* *0.41; two-way ANOVA; Fig. 2*G*,*H*). In light of the observed reduction in mIPSC frequency, the increased PPR of PV-mediated IPSCs suggests that there is a presynaptic deficit in GABA release from PV cells to L2/3 PNs in the mPFC of *Disc1* LI mice.

### Reduced feedforward inhibition (FFI) in a thalamus–mPFC circuit in *Disc1* LI mice

The mediodorsal nucleus of the thalamus (MD) sends major projections to the mPFC. This MD–mPFC circuit has been implicated in cognitive processes such as WM (Parnaudeau et al., 2013, 2018; Bolkan et al., 2017; Ferguson and Gao, 2018b) and cognitive flexibility (Parnaudeau et al., 2015; Rikhye et al., 2018) that are impaired in schizophrenia (Lesh et al., 2011; Parnaudeau et al., 2013, 2015). We and others have shown that excitatory inputs from the MD drive mPFC FFI (Miller et al., 2017; Collins et al., 2018; Meda et al., 2019) that is primarily mediated by mPFC PV INs (Delevich et al., 2015). Given the deficit in GABA release from PV INs to PNs in the mPFC of *Disc1* LI mice (Fig. 2), we reasoned that FFI in the MD–mPFC circuit may be affected in these mice. To test this hypothesis, we injected the MD of *Disc1* LI mice and their WT littermates with AAV-ChR2(H134R)-YFP. After viral expression reached sufficient levels, we used these mice to prepare acute brain slices, in which we recorded both excitatory and inhibitory synaptic transmission onto dACC L3 PNs in response to optogenetic stimulation of MD axons (Fig. 3*A*,*B*).

Brief (0.5 ms) light stimulation evoked monosynaptic EPSCs and disynaptic IPSCs onto L3 PNs in the dorsal mPFC (Fig. 3*C*; and see Delevich et al., 2015). We found that the contribution of inhibitory synaptic transmission to total synaptic inputs, measured as IPSC^peak^/(IPSC^peak^+EPSC^peak^), or I^peak^/(I^peak^+E^peak^), was significantly lower in *Disc1* LI mice than in WT mice when comparing the means of the two groups of animals (*Disc1* LI, 0.52 ± 0.03, *N* = 11 mice; WT, 0.70 ± 0.02, *N* = 14 mice; *t*_(23)_ = 5.73, ^j^*p *<* *0.0001, *t* test; Fig. 3*D*), or the means of the two groups of neurons (*Disc1* LI, 0.60 ± 0.03; *n* = 30 cells, WT, 0.70 ± 0.02, *n* = 40 cells; *t*_(68)_ = 3.17, ^k^*p *<* *0.01, *t* test; Fig. 3*E*). In addition, the slope of a linear regression describing the relationship between IPSC and EPSC peak amplitudes of individual neurons in the *Disc1* LI mice was significantly lower than that in WT (Fig. 3*F*). The latencies (Fig. 3*G–I*) and kinetics of the EPSCs (Fig. 3*J*,*K*) and IPSCs (Fig. 3*L*,*M*) in *Disc1* LI mice were similar to those in WT mice. These results together indicate that *Disc1* LI is associated with reduced MD-driven FFI in the mPFC.

### Spontaneous excitatory synaptic transmission onto PV INs and their intrinsic properties are unchanged in *Disc1* LI mice

The decrease in FFI in the MD–mPFC pathway in *Disc1* LI mice could result from the impairment in GABA release from PV INs in the mPFC (Fig. 2), or reduced recruitment of mPFC PV INs by MD. Thalamically-driven FFI relies on the ability of PV INs to reach threshold in response to thalamic inputs, a process dependent on both synaptic and intrinsic properties of PV INs. DISC1 is expressed in MGE-derived inhibitory INs including PV INs (Schurov et al., 2004; Meyer and Morris, 2008; Steinecke et al., 2012; Seshadri et al., 2015) raising the possibility that *Disc1* LI could alter excitatory synaptic transmission onto PV INs and/or their intrinsic properties. We crossed the *Disc1* LI mouse line onto the *PV-Cre::tdTomato* lines, allowing us to assess the synaptic and intrinsic properties of visually identified PV INs in the context of *Disc1* LI. We found that mEPSC amplitude and frequency onto mPFC PV INs was consistent between genotypes (amplitude: WT, 12.39 ± 0.40 pA; *Disc1* LI, 13.48 ± 0.45 pA, *n* = 20, 23 cells/genotype, *N* = 4 mice/genotype; *t*_(41)_ = 1.78, ^l^*p* = 0.08; frequency: WT, 6.99 ± 0.74 Hz; *Disc1* LI, 6.55 ± 0.69 Hz, *t*_(41)_ = 0.53, ^m^*p *=* *0.60; Fig. 4*A*,*B*). Next, we examined the intrinsic properties of PV INs in WT and *Disc1* LI mice and found no significant differences between genotypes (Fig. 4*C–H*), including minimum current injection required to elicit spiking (WT, 142.3 ± 12.67 pA; *Disc1* LI, 119.2 ± 14.3 pA, *t*_(24)_ = 1.21, ^n^*p* = 0.24; Fig. 4*G*) or maximum firing rate (WT, 88.92 ± 3.9 Hz; *Disc1* LI, 88.92 ± 6.78 Hz, *t*_(24)_ = 0, ^o^*P *>* *0.99; Fig. 4*H*). These results suggest that neither intrinsic excitability of prefrontal PV INs nor spontaneous glutamatergic transmission onto them is grossly perturbed in *Disc1* LI mice.

### Enhanced input but reduced output of PV INs in *Disc1* LI mice

**Figure 5.**
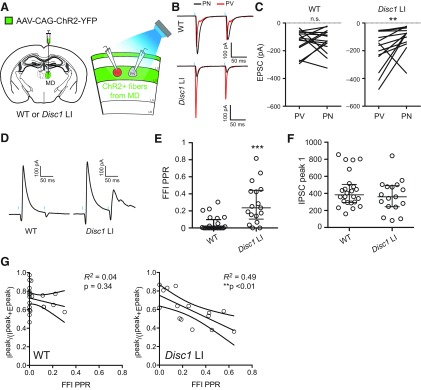
Altered presynaptic function of PV INs underlies the deficit of FFI in *Disc1* LI mice. ***A***, left, schematic of the experimental configuration. Right: schematic of the recording configuration in the mPFC acute slices. A Tdtomato^+^ PV IN (red) and an adjacent PN (gray) in L3 of the mPFC were recorded simultaneously or sequentially. EPSCs onto these neurons were evoked by optogenetic stimulation (0.5-ms light pulses; blue bars) of MD axons. ***B***, Sample EPSC traces recorded from PV IN and PN pairs are superimposed and color-coded. ***C***, Quantification of the EPSC peak amplitude. n.s., not significant (*p *=* *1.0); **p <* *0.01; Wilcoxon matched-pairs signed ranks test. ***D***, Sample traces of FFI currents recorded from L3 PNs in response to optogenetic stimulation of MD axons. ***E***, Quantification of PPR of the MD-driven FFI onto L3 PNs; ***p <* *0.001, Mann–Whitney *U* test. ***F***, The mean amplitude of the first IPSC was consistent between genotypes. ***G***, FFI PPR plotted against (I^peak^)/(I^peak^ + E^peak^; as seen in Fig. 3*D*) within cells shows that for *Disc1* LI mice but not WT mice, FFI PPR inversely correlated with (I^peak^)/(I^peak^ + E^peak^), suggesting that synapses with high FFI PPR also exhibit higher E/I ratio; **p < 0.01. Data in ***E*** presented as median ± interquartile range data in ***F*** presented as mean ± SEM.

We next examined recruitment of mPFC PV INs specifically within the MD–mPFC circuit to determine if reduced excitatory drive could account for the observed reduction in FFI in *Disc1* LI mice. We recorded evoked EPSCs onto PV IN and PN pairs in the mPFC in response to optogenetic stimulation of MD axons (Fig. 5*A*). We found that in WT mice, amplitudes of thalamocortical EPSCs were similar between PV INs and neighboring PNs (PV, −109.3 ± 109.7 pA, PN, −129.1 ± 103.2 pA, *n* = 15 pairs, *W* = 0, ^p^*p *=* *1.0, Wilcoxon matched-pairs signed rank test; Fig. 5*B*,*C*). By contrast, in *Disc1* LI mice, thalamocortical EPSCs onto PV INs were much larger than those onto neighboring PNs (PV, −153.4 ± 211.9 pA; PN, −73.74 ±104.6 pA; *n* = 14 pairs, W = −83, ^q^*p *<* *0.01 Wilcoxon matched-pairs signed rank test; Fig. 5*B*,*C*). These data suggest that MD excitatory drive onto PV INs is enhanced relative to L2/3 PNs in the mPFC of *Disc1* LI mice. Therefore, reduced excitatory synaptic strength onto PV INs does not account for the decrease in FFI in the MD–mPFC circuit in *Disc1* LI mice compared with WT (Fig. 3).

Next, we probed presynaptic GABA release from PV cells within the MD–mFPC circuit, by optogenetically stimulating the MD axons (see the recording configuration in Fig. 3*A*,*B*) and measuring the PPR of MD-driven FFI onto mPFC PNs (Fig. 5*D*). Notably, we found that PPR of was significantly higher in *Disc1* LI mice than in WT mice (WT: 0.0 ± 0.1, *n* = 24 cells, *N* = 10; *Disc1* LI: 0.24 ± 0.34, *n* = 17 cells, *N* = 6; *U *= 62, ^r^*p *=* *0.0001, two-tailed Mann–Whitney *U* test; Fig. 5*D*,*E*), mirroring the increase in PPR we observed when directly activating PV INs (Fig. 2*C*). In order to reduce variability in measuring the PPR, we set the light-stimulation such that there was no difference between genotypes in the average amplitude of the first evoked IPSCs (WT, 439.3 ± 41.31 pA, *n* = 24 cells, *N* = 10; *Disc1* LI, 367.1 ± 46.26 pA, *n* = 17 cells, *N* = 6; *t*_(39)_ = 1.152, ^s^*p *=* *0.256, unpaired *t* test; Fig. 5*F*). Finally, we examined the relationship between PPR of MD-driven FFI and E-I ratio of MD-driven synaptic currents onto PNs. We found that there was a significant inverse correlation between FFI PPR and I^peak^/(I^peak^+E^peak^) within PNs from *Disc1* LI mice but not WT mice (Fig. 5*G*). Together, our data suggest that in *Disc1* LI mice, GABA release from prefrontal PV INs is reduced, leading to decreased FFI in the MD–mPFC circuit.

## Discussion

Perturbation of the multifunctional scaffolding protein DISC1 is linked to a range of behavioral phenotypes that are associated with major psychiatric disorders (Brandon and Sawa, 2011). These findings highlight DISC1 as a promising molecular lead to investigate the molecular pathways and neural circuits that underlie major mental illnesses (Niwa et al., 2016). Here, we used the *Disc1* LI mouse model to investigate the function of mPFC circuits that may be particularly relevant to the cognitive symptoms of psychiatric disorders. We found that *Disc1* LI mice exhibited elevated E-I ratio, measured as a reduction of spontaneous inhibitory transmission onto L2/3 PNs in mPFC and decreased FFI onto L2/3 PNs in the MD–mPFC circuit. Several lines of evidence suggest that this effect can be accounted for by a reduction in GABA release from PV INs in the mPFC: 1) mIPSC frequency was significantly reduced onto L2/3 PNs in *Disc1* LI mice, consistent with a reduction in presynaptic release probability; 2) the PPR of IPSCs directly evoked by optogenetic stimulation of PV INs but not SOM INs was significantly increased in *Disc1* LI mice compared with WT; and 3) the PPR of MD-evoked FFI, which is almost exclusively driven by PV INs under the experimental conditions used (Delevich et al., 2015), was increased in *Disc1* LI mice and correlated with E-I ratio. Together, our findings suggest that PV **→** PN synapses are the primary site of impairment in the MD–mPFC circuit in *Disc1* LI mice.

It has been hypothesized that the cognitive deficits in psychiatric diseases may be the consequence of imbalanced excitation and inhibition (E-I) in key neural circuits (Kehrer et al., 2008; Lisman, 2012; Marin, 2012; Krystal et al., 2017; Ferguson and Gao, 2018a). Consistent with this hypothesis, several studies have shown that experimentally imposing elevated E-I within the mPFC impairs cognitive processing in rodents (Yizhar et al., 2011; Cho et al., 2015; Murray et al., 2015; Ferguson and Gao, 2018b). In addition to evidence of altered PV IN mediated inhibition, we observed that excitatory synaptic transmission onto PV cells driven by MD inputs was enhanced in *Disc1* LI mice, which could compensate for presynaptic deficits in PV IN function. Indeed, a recent study that examined multiple autism genetic mouse models found that increased E-I ratio did not drive network hyperexcitability but in fact led to homeostatic stabilization of excitability (Antoine et al., 2019). Therefore, potential network effects arising from altered E-I conductance ratios should not be over interpreted, and it remains unclear how the changes we observed in the *Disc1* LI mice affect network activity *in vivo* and resulting behavior.

In humans, *Disc1* polymorphisms are associated with measures of cognitive performance and frontal lobe structure among some ethnic groups (Burdick et al., 2005; Cannon et al., 2005; Hennah et al., 2005; Liu et al., 2006; Palo et al., 2007; Carless et al., 2011; Nicodemus et al., 2014). Multiple mouse models of DISC1 perturbation exhibit cognitive impairments (Koike et al., 2006; Clapcote et al., 2007; Li et al., 2007; Kvajo et al., 2008; Lipina et al., 2010; Niwa et al., 2010; Brandon and Sawa, 2011; Lee et al., 2013), strengthening the mechanistic link between DISC1 and cognition. Here we provide evidence of cell type-specific alterations within mPFC circuits implicated in multiples aspects of cognition. While we did not examine cognition in the *Disc1* LI mice, *Disc1* LI (−/−) mice are reported to exhibit blunted startle response and prepulse inhibition (PPI; Jaaro-Peled et al., 2018), a behavior that is regulated by the mPFC (Swerdlow et al., 2001; Schwabe and Koch, 2004). Future experiments interrogating mPFC-dependent cognition in *Disc1* LI mice will be critical for relating the circuit-level changes we observed in E-I balance and PV IN function to behavior.

While our study is the first to specifically detect a presynaptic deficit in PV INs in a DISC1 genetic deficiency model, previous studies using different transgenic models have reported that DISC1 influences inhibitory IN function or development: spontaneous IPSC frequency is reduced in the frontal cortex of male mice expressing truncated mouse DISC1 (Holley et al., 2013); PV IN function is impaired in the mPFC of mice overexpressing a truncated form of DISC1 (Sauer et al., 2015); PV expression is reduced in the PFC of several *Disc1* mouse models (Hikida et al., 2007; Shen et al., 2008; Niwa et al., 2010; Ayhan et al., 2011; Lee et al., 2013); and tangential migration of MGE-derived neurons is impaired by *Disc1* mutation or RNA interference (Steinecke et al., 2012; Lee et al., 2013). These findings provide converging evidence that DISC1 perturbation alters PFC inhibition. Our current findings more specifically implicate the presynapse of PV INs as a site of impairment in mice harboring a *Disc1* LI allele, which is the most extensively perturbed form of the gene reported to date (Shahani et al., 2015).

Several important caveats should be considered when interpreting our electrophysiology results. First, a reduction in mIPSC frequency is also consistent with a reduced number of inhibitory synapses. A recent study investigating *Disc1* LI mice reported no change in the number of PV INs themselves within the mPFC (Seshadri et al., 2015). In addition, we focused on PV and SOM IN function, which together comprise ∼70% of cortical INs (Rudy et al., 2011). It is therefore possible that the remaining 30% of IN cell types, e.g., 5HT3a receptor-expressing neurons, also contribute to the reduced mIPSC frequency observed in *Disc1* LI mice. Next, IPSCs directly evoked by optogenetic stimulation significantly overlapped at short interstimulus intervals; therefore, changes in the input resistance due to open channels likely influenced the size of the second signal and hence the PPR measurement. More detailed analysis such as multiple probability-compound binomial analysis or direct analysis of failure rate is necessary to conclude that GABA release probability from PV INs is reduced in *Disc1* LI mice. We observed that PV IN evoked IPSC PPR was significantly increased at the 50- and 100-ms interstimulus intervals but not at the 150-ms interval. The time dependence of this effect may suggest a postsynaptic mechanism, such as GABA-mediated regulation of PPR (Kirischuk et al., 2002). Alternatively, GABA_B_ presynaptic regulation may play a role in influencing PPR. Interestingly, a reduction in GABA_B_ receptor expression in PNs has been observed in postmortem brain tissue of individuals with schizophrenia (Mizukami et al., 2002). These caveats considered, we provide multiple lines of evidence that are consistent with elevated E-I balance and abnormal PV IN function in mPFC circuits in *Disc1* LI mice.

The coordinated activity between the MD and the PFC is important for WM, attention, and flexible goal-oriented behavior (Mitchell and Chakraborty, 2013; Parnaudeau et al., 2013, 2015; Schmitt et al., 2017; Alcaraz et al., 2018), faculties that are impaired in a variety of psychiatric disorders. Meanwhile, studies have found that MD–mPFC synaptic strength is modulated by social interaction, perhaps relevant to negative symptoms of schizophrenia and depression (Northoff and Sibille, 2014; Franklin et al., 2017; Zhou et al., 2017). In relation to DISC1, one study found that a common missense variant of *Disc1* was associated with altered thalamofrontal functional connectivity (Liu et al., 2015). Notably, patients with schizophrenia and bipolar disorder exhibit reduced MD-PFC functional connectivity relative to healthy controls (Welsh et al., 2010; Woodward et al., 2012; Anticevic et al., 2014). An emerging hypothesis posits that local disinhibition of PFC may destabilize the flow of information through the thalamofrontal loop and contribute to cognitive and negative symptoms in schizophrenia and related disorders (Anticevic et al., 2012; Murray and Anticevic, 2017). Structural alterations within thalamofrontal circuits have also been linked to cognitive deficits associated with aging (Hughes et al., 2012) and epilepsy (Pulsipher et al., 2009). However, until recently there was a paucity of data describing how the MD and frontal cortex interact at the neural circuit level. Recent studies have demonstrated that the MD thalamus recruits FFI in the rodent mPFC (Delevich et al., 2015; Miller et al., 2017; Collins et al., 2018; Meda et al., 2019) that is primarily mediated by PV INs (Delevich et al., 2015). Interestingly, chemogenetic excitation of mPFC PV INs has been shown to rescue cognitive deficits induced by chemogenetic inhibition of MD (Ferguson and Gao, 2018b).

Our findings extend data suggesting that MD, via its projections to PV INs, is a key regulator of E-I balance that underpins PFC circuit function. We demonstrate that reduced DISC1 expression, a key molecular candidate to study biology relevant to behavioral constructs related to several psychiatric disorders, leads to elevated E-I balance in the MD–mPFC thalamofrontal circuit. Given that few treatment options exist to address the cognitive symptoms of psychiatric disorders, efforts towards understanding the cellular and molecular mechanisms underlying abnormal thalamofrontal functional connectivity may yield therapies that will improve patient outcomes.

## Synthesis

Reviewing Editor: Karen Szumlinski, University of California at Santa Barbara Decisions are customarily a result of the Reviewing Editor and the peer reviewers coming together and discussing their recommendations until a consensus is reached. When revisions are invited, a fact-based synthesis statement explaining their decision and outlining what is needed to prepare a revision will be listed below.

Upon consultation with two expert reviewers, we have some to the decision of “Revise-Editorial Review Only” regarding your recent submission to eNeuro. All three of us agree that the report was very well-written, the data presented in a clear and concise manner. The Discussion, although lacking in some detail as highlighted by Reviewer 1 below, was thorough and well thought-out. Both reviewers and I agree that this report brings light to the regulation of MD-dAcc communication of relevance to neuropsychiatric phenotypes and we all agree that it is highly relevant to the readers of eNeuro. I have copied below the comments from both reviewers. As noted below, Reviewer 1 has some issues that we all agree should be addressed in a revised version of this report. I am happy to re-review this revision without further need for additional peer re-review.

Reviewer 1:

Manuscript eN-NWR-0496-19 looks at changes in inhibition in the prefrontal cortex (PFC) of mice heterozygous for a deficiency allele of the Disc1 gene (Disc1df/+). Changes in PFC excitation/inhibition balance are associated with behavioral deficits, and DISC1, although no longer considered a valid common risk gene for schizophrenia, has been raised as a tool for the study of endophenotypes of mental disorders. The authors used electrophysiology, viral and transgenic approaches to assess inhibition driven by parvalbumin (PV) and somatostatin (SOM) interneurons (INs), as well as mediodorsal thalamus projections onto PFC principal neurons (PNs). They saw that Disc1df/+ principal neurons in layer2/3 of dorsal ACC (dACC) had a reduction in mIPSC frequency at P70 and P15. Similarly, using PV or SOM-Cre animals and injection of cre-dependent AAV-ChR2 in PFC, they found an increase in paired pulse ratios derived from stimulation of PV (but not SOM) interneurons into L2/3 PNs in Disc1df/+ mice, consistent with a decrease in GABA release probability from PV interneurons onto PNs.

The next part of the manuscript is interested in characterizing the mediodorsal thalamus (MD) inputs to prefrontal cortex in Disc1df/+ mice, as this pathway has been implicated in working memory and cognitive flexibility deficits which are present in schizophrenia. They saw that MD-dACC relative inhibition was reduced in Disc1df/+ mice, consistent with a reduction in feedforward inhibition onto dACC PNs in this pathway. When they patched PV interneurons, they saw no difference in mEPSCs or intrinsic properties, indicating the deficit in feedforward inhibition is likely not being driven by changes in PV excitability. Using paired recordings and optogenetics, they also found that MD input into PV INs was larger than that to PNs in Disc1df/+ mice, which was equivalent for both cell types in wild type. They saw a similar increase in paired pulse ratios for MD-induced feedforward inhibition onto dACC PNs in Disc1df/+ mice, and a correlation between MD-evoked relative inhibition and FFI PPR in the mutants. Their data points to Disc1df/+ mice showing a general reduction in PV GABA release in dACC, contributing to a reduction in MD-dACC feedforward inhibition.

The manuscript conducts a very interesting and thorough characterization of inhibition in the dACC of Disc1df/+ mice. The cell-specific manipulations and MD-dACC experiments add important insight to the micro and some of the macrocircuit changes driven by this deletion. If accompanied by equivalent behavioral deficits in these animals, this would add important information to our understanding of the contribution of these circuits and DISC1 to cognitive function.

Major concerns:

My main concern relates to the framing and impact of the results. The authors provide a confusing rationale for the impact of this work. They acknowledge that DISC1 is no longer considered a major risk factor for schizophrenia, yet frame their MD-dACC experiments as pertinent to schizophrenia. They also say DISC1 seems to be interesting in relation to development, yet mostly look in adulthood. Clearer and more targeted background pertinent to the effects of DISC1 (and particular their DISC1 deletion) to the clinic and behavior would strengthen the manuscript. At present, their results are not directly anchored in the broader literature, and assessing their impact is challenging.

Specifically, the authors mention looking at the MD-dACC pathway due to its involvement in working memory and cognitive flexibility. However, it is not mentioned whether the Disc1df/+ mice show deficits in these behaviors. Have these mice been phenotyped at all in terms of behavior? How may these changes in physiology contribute to behavioral deficits, and how is that relevant to DISC1-related behavioral deficits in the clinic?

More detail on the precise timeline of viral infusion and age of the animals should be provided. Did experimental groups include animals with different delays between surgery and recording? In the methods, the authors state a delay of 2-4 weeks took place. The delay should be identical for all experiments, as it may affect synaptic responses. For ratios this is less of a concern, however it should be stated and ideally comparable between and within groups.

Minor concerns:

Line 306: “3)2)” - the 2) should be removed

Reviewer 2:

This manuscript details network function in layer 2/3 principal neurons of the medial prefrontal cortex and its input from the mediodorsal thalamus, and reveals dysregulation of parvalbumin interneuron function that occurs in Disc1 deficient mice. This work expands on recent studies investigating the impact of input from the MDThal on mPFC function. The manuscript is well-written, analyses conducted appropriately, and limitations recognized. The inclusion of a number of diverse synaptic properties reveals increased understanding of the MDThal-mPFC cirucit in both wild-type mice and identifies a precise impairment in the output of parvalbumin neurons in a relevant mouse model for psychiatric illness. I have no recommendations or comments that I would deem necessary in needing to be addressed and find the findings exciting and opening a diverse number of avenues for future investigation.
